# The Prophylactic and Therapeutic Effects of Fermented* Cordyceps sinensis* Powder, Cs-C-Q80, on Subcortical Ischemic Vascular Dementia in Mice

**DOI:** 10.1155/2018/4362715

**Published:** 2018-12-18

**Authors:** Ying Chen, Lifeng Fu, Min Han, Meiying Jin, Jiaying Wu, Li Tan, Zhong Chen, Xiangnan Zhang

**Affiliations:** ^1^Department of Ophthalmology, Affiliated Sir Run Run Shaw Hospital, School of Medicine, Zhejiang University, Hangzhou, China; ^2^Institute of Pharmacology and Toxicology, College of Pharmaceutical Sciences, Zhejiang University, Hangzhou, China; ^3^Hangzhou Zhongmei Huadong Pharmaceutical Co., Ltd., Hangzhou, China

## Abstract

Corbrin Capsule, a preparation of* Cordyceps sinensis* analogue, is a pleiotropic traditional Chinese patent medicine with the main component of fermentative cordyceps fungus powder (Cs-C-Q80). The neuroprotective effects of Cs-C-Q80, as a substitution of* Cordyceps sinensis*, have not been fully identified. The objectives of this study were to explore the prophylactic and therapeutic effects of Cs-C-Q80 in vascular dementia mice model. The efficacy of Cs-C-Q80 was investigated in a molecular level as well. The subcortical ischemic vascular dementia was modelled by permanent right unilateral common carotid arteries occlusion (rUCCAO) in adult male mice. The animals were randomly divided and treated by gavage with vehicle (1% CMC-Na solution) (rUCCAO model) or Cs-C-Q80 powder at 0.2 g/kg or 1.0 g/kg, respectively. Preventive treatment was administrated by gavage daily for 7 days before rUCCAO, while therapeutic treatment was administrated continuously from 28 days after rUCCAO. Object recognition test and Morris water maze test were performed to evaluate the learning and working memory. The luxol fast blue stain (Kluver-Barrera method) and immunohistochemistry for myelin basic protein (MBP) were employed to determine the severity of white matter damage. Both preventive and therapeutic treatment with Cs-C-Q80 protected against the rUCCAO-induced memory impair in mice as determined by object recognition and Morris water maze tests. The histopathological analyses revealed significant white matter rarefaction and reduction of MBP expression in corpus callosum after rUCCAO, which could be counteracted by either preventive or therapeutic treatment with Cs-C-Q80. Moreover, the Cs-C-Q80 treatments inhibited rUCCAO-induced astrocytes activation and the tumor necrosis factor *α* (TNF-*α*) and interleukin-1*β* expression, indicating the anti-inflammatory roles of Cs-C-Q80 against subcortical ischemia. Cs-C-Q80 is a potential preparation for the prophylaxis and treatment of subcortical ischemic vascular dementia. The underlying pharmacological efficacy might be associated with suppression of myelin degeneration, glia activation, and inflammatory cytokines release.

## 1. Introduction

Vascular dementia, the second most common type of dementia, is caused by insufficient blood supply to the brain resulted from small-artery disorder. Subcortical ischemic vascular dementia (SIVD) is a main cause of vascular dementia and cognitive impairment in elderly people [1, 2]. The typical pathological changes of SIVD are the development of ischemic white matter damage, which consists of vacuolation, demyelination, axonal loss, and lacunar infarcts [3]. Chronic cerebral hypoperfusion induces white matter injury and dementia in mice. The mice undergo right unilateral common carotid arteries occlusion (rUCCAO). This model recapitulates the pathological and behavioral features of SIVD observed in human [2, 4]. Pathological studies have demonstrated the markers of oxidative stress and inflammation in the impaired white matter from vascular dementia patients. Unfortunately, however, there are no FDA-approved treatments for vascular dementia.

*Cordyceps sinensis (Berk) Sacc.* is a valuable entomogenous fungus used as a traditional Chinese medicine to replenish health. The efficacy of* Cordyceps sinensis* extracts in cellular and molecular levels has been expanded to immunostimulating, free radicals scavenging, antisenescence, and endocrine regulations [5]. Recently,* Cordyceps sinensis* and its derivations are regarded as the potent neuroprotective agents against acute brain injury [6–10], but the effects on vascular dementia have not been determined. Moreover, the clinical application of* Cordyceps sinensis *was restricted for harsh cultivation surroundings, low yield, and exorbitant price. Cs-C-Q80 is an ideal substitution for natural* Cordyceps sinensis *[11, 12]. Cs-C-Q80, a preparation of* Cordyceps sinensis* analogue, is a pleiotropic traditional Chinese patent medicine. The aim of this study was to explore the prophylactic and therapeutic effects of Cs-C-Q80 on SIVD and to provide evidence to support its future registration for new indications such as vascular dementia.

## 2. Materials and Methods

### 2.1. Reagents and Antibodies

The Cs-C-Q80 was offered by Huadong Pharmaceutical Co., Ltd. (Hangzhou, China), with production Lot. no. of 130301. The mouse monoclonal antiglial fibrillary acidic protein (anti-GFAP) was purchased from Sigma-Aldrich (St. Louis, MO, USA). The mouse monoclonal antibody anti-glyceraldehyde-3-phosphate dehydrogenase (anti-GAPDH) was from Boster (Wuhan, Hubei, China). The rat monoclonal antimyelin basic protein (anti-MBP) was from Millipore (Billerica, MA, USA). The antitumor necrosis factor *α* (TNF-*α*) was product of R&D (Minneapolis, MN, USA). The anti-interleukin 1*β* (IL-1*β*) was product of Abcam (Cambridge, UK). The second antibodies were the products of Multi Sciences (Shanghai, China).

### 2.2. Animal Preparation and Treatment

**Figure 1:**
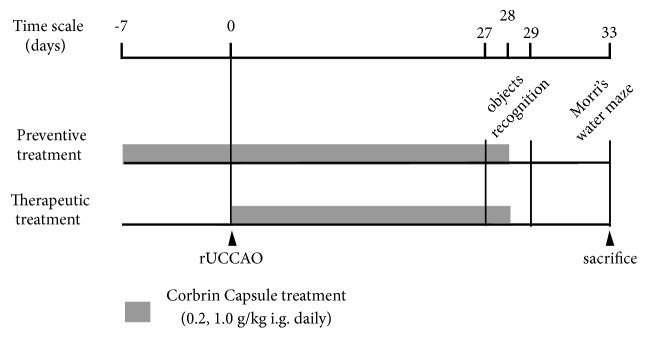
The schedule for administration and experiments.

The surgical procedure of rUCCAO was performed on eight to ten-week-old male ICR mice weighing 22 to 25 g as previously described [2]. These mice were bred in a specific pathogen-free facility with 4-6 mice in every cage with controlled temperature (27°C) and humidity (65%). They were access to food and water freely. The light/dark cycle was set as 12/12 hr with light on from 8:00 to 20:00. The animals were randomly divided into groups following a random number table generated by Excel software. LF, MH, and MJ from the authors randomly divided the animals and were blinded to the groups when they analyzed the data. Briefly, the mice were anesthetized by inhaling 2.5% isoflurane and oxygen mixed gas during surgery; the right common carotid artery of mice was isolated and double-ligated with 6-0 nylon monofilament. The powder of Cs-C-Q80 was suspended in 1% sodium carboxymethylcellulose (CMC-Na) solution. To study the prophylactic effects, mice were administrated with Cs-C-Q80 (0.2 g/kg, 1.0 g/kg or vehicle) by gavage daily for 7 days before the rUCCAO surgery and the administration was continued for another 28 days after surgery. To study the therapeutic effects, mice were administrated continuously for 28 days after rUCCAO surgery in the same way. As shown in Figure 1, on Day 27 after rUCCAO, the animals were subjected to object recognition test for present and on Day 28 for test procedure. The 5-day Morris water maze test started from Day 29 and ended on Day 33. On Day 33 after rUCCAO, the mice were sacrificed and the brains were removed for immunohistochemical staining and other examinations. Sham mice were given the same volume of 1% CMC-Na solvent (daily,* i.g.*) and subjected to the same surgical procedure without carotid ligation. The experiments were conducted under a protocol approved by the Institutional Animal Care and Use Committee of Zhejiang University (Permission Document no. ZJU20150714).

### 2.3. Object Recognition Test

The object recognition test was performed to assess nonspatial working memory linked to frontal-subcortical circuits [13]. A glass box (30×45×30 cm) was used as the experimental apparatus. The objects to be discriminated were plastic and were in three different shapes and colors of 5.8 cm height. On the first day, the mice were allowed to explore the box without any objects for 10 min. On the second day, two sections of trial were carried out. The interval of experiment was 1 h. In the first section, two uniform objects appeared on two opposite sides of the box, and the mice were allowed to explore for 10 min. Exploration was defined as directing the nose to the object at a distance <1 cm and/or touching it with the nose. In the second section, one of the objects presented previously was substituted with another novel object. The observation time for the mice was shortened to 3 min. The discerning time for the familiar (F) objects and the new (N) ones were recorded manually by someone who was blinded to this experiment. Discrimination index was calculated by (N-F/N+F)×100% as the indicator among groups.

### 2.4. Morris Water Maze Test

The Morris water maze was performed to evaluate the spatial learning and memory [14]. The circular tank was 150 cm in diameter and 50 cm in height with nonreflective interior surfaces and was filled with water at a depth of 30 cm, so that the mice could neither escape over the edge of the tank nor balance its tail on the bottom of the tank. The platform was 10 cm in diameter and was submerged 1.5 cm below the surface of the water. Mice were released into the water randomly in different quadrants at water-level. Our experimental design was 4 trials of the first day and 6 trails for the next two days for the acquisition phase, and the interval between the trials was 10 min. For each trial, the escape latency was defined as the time to reach the platform and climb up out of the water, which was limited to 1 min. And then, the average escape latency for each day was analyzed.

### 2.5. Histopathology and Immunohistochemistry

Thirty-three days after Sham or rUCCAO operation, mice were sacrificed and the brains tissues were isolated and fixed in 4% paraformaldehyde at 4°C for 24 h, and then in 30% sucrose for 3 days. Frozen brain sections (10 *μ*m-thick) were cut using a cryostat (SM2000R, LEICA, Wetzlar, Germany).

The luxol fast blue stain (Kluver-Barrera method) was employed to evaluate the fiber density in the corpus callosum which is an index for white matter lesions [15]. Brain sections were incubated with PBS containing 3% normal donkey serum, 0.3% Triton X-100 for 2 h, and then with the primary antibodies (anti-GFAP 1:500 and anti-MBP 1:250) overnight at 4°C. The sections were then incubated with FITC-conjugated anti-rat IgG antibody for MBP; FITC-conjugated anti-mouse IgG antibody for GFAP (1:500) for 2 h at room temperature. Finally, the slices were visualized by a fluorescence microscope (Olympus BX51, Tokyo, Japan).

### 2.6. Western Blot

The isolated corpus callosum was homogenized in RIPA buffer (Tris-HCl 20 mM, pH 7.5, NaCl 150 mM, EDTA 1mM, 1% Triton X-100, 0.5% sodium deoxycholate, PMSF 1 mM, and leupeptin 10 *μ*g/ml). An aliquot of 40 *μ*g protein from each sample was separated by SDS-PAGE and transferred to a nitrocellulose membrane under standard protocols. Primary antibodies were incubated at 4°C overnight. GAPDH was used as house-keeping proteins for brain tissues. Secondary antibodies were incubated for 2 h at room temperature. The blots were visualized by an Odyssey infrared imaging system (LI-COR Biosciences, 9120) and analyzed using the Odyssey software.

### 2.7. Statistical Analysis

Data are presented as mean ± standard error of mean (SEM). Statistical analysis was performed with SPSS 11.5 for Windows (SPSS Inc. Chicago, IL, USA). One-way analysis of variance (ANOVA) followed by the Turkey's post hoc test was used for multiple comparisons. The escape latency in the Morris water maze test was analyzed by two-way ANOVA followed by the LSD test.* P* value<0.05 was considered to be statistically significant.

## 3. Results

### 3.1. Prophylactic Administration of Cs-C-Q80 Attenuated Spatial and Working Memory Impairment Induced by rUCCAO

**Figure 2:**
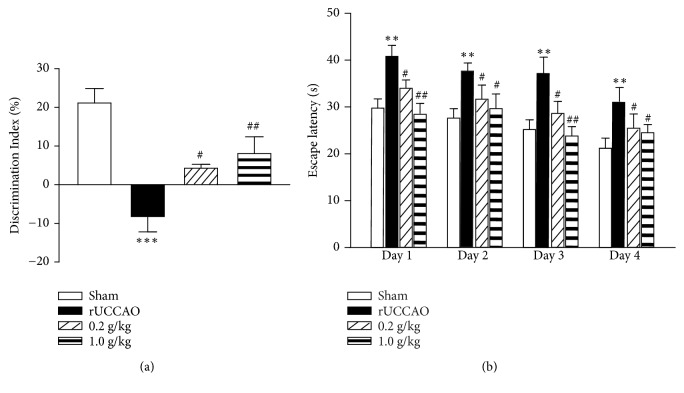
**The effects of prophylactic administration of Cs-C-Q80 on object recognition and Morris water maze after rUCCAO in mice.** Mice were administrated with indicated dosage of Cs-C-Q80 7 days before modelling of rUCCAO and continuously for next 28 days. (a) The object recognition test and (b) the Morris water maze test were performed from 27 to 33 days after rUCCAO. Values are represented as mean ± SEM.*∗∗ P*<0.01; *∗∗∗P*<0.001* versus* the Sham group; #*P*<0.05; ##*P*<0.01* versus* the rUCCAO group, n=9.

The nonspatial working memory was evaluated by object recognition test. As shown in Figure 2(a), rUCCAO-treated mice presented a remarkable decrease in discrimination index compared with Sham (*P*<0.001), implying the seriously impaired discrimination ability. Either 0.2 g/kg or 1.0 g/kg Cs-C-Q80 pretreatment before the rUCCAO relieved the cognitive impairment in a dose-dependent manner. As shown in Figure 2(b), in Morris water maze test, prophylactic administration of Cs-C-Q80 significantly reduced the escape latencies [F(3,96)=10.23,* P*<0.0001]. These results revealed that prophylaxis with Cs-C-Q80 improves both the nonspatial working memory and spatial learning memory in mice which suffered from rUCCAO.

### 3.2. Therapeutic Administration of Cs-C-Q80 Attenuated Spatial and Working Memory Impairment Induced by rUCCAO

**Figure 3:**
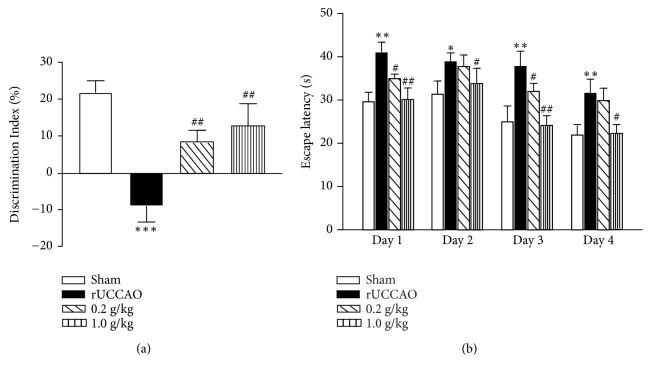
**The effects of therapeutic administration of Cs-C-Q80 on object recognition and Morris water maze after rUCCAO in mice.** Mice were administrated with indicated dosage of Cs-C-Q80 after the onset of rUCCAO for 28 days. (a) The object recognition test and (b) the Morris water maze test were performed from 27 to 33 days after rUCCAO. Values are represented as mean ± SEM. *∗P*<0.05; *∗∗P*<0.01; *∗∗∗P*<0.001* versus* the Sham group; #*P*<0.05; ##*P*<0.01* versus* the rUCCAO group; n=9.

We next investigated the therapeutic effects of Cs-C-Q80 against rUCCAO injuries. As shown in Figure 3(a), the discrimination index in object recognition test was decreased to -9% of the rUCCAO mice from the 22% of Sham group. Either 0.2 g/kg or 1.0 g/kg Cs-C-Q80 treatment for 28 days after rUCCAO significantly improved the discrimination index in a dose-dependent manner (*P*<0.01). In addition, in Morris water maze test, after treatment with Cs-C-Q80 for nearly a month, there was significant reduction in the escape latencies [F(3,96)=12.48,* P*<0.0001] suggesting the tangible efficacy of Cs-C-Q80 administration for vascular dementia.

### 3.3. Both Prophylactic and Therapeutic Application of Cs-C-Q80 Ameliorated the White Matter Damage after rUCCAO

**Figure 4:**
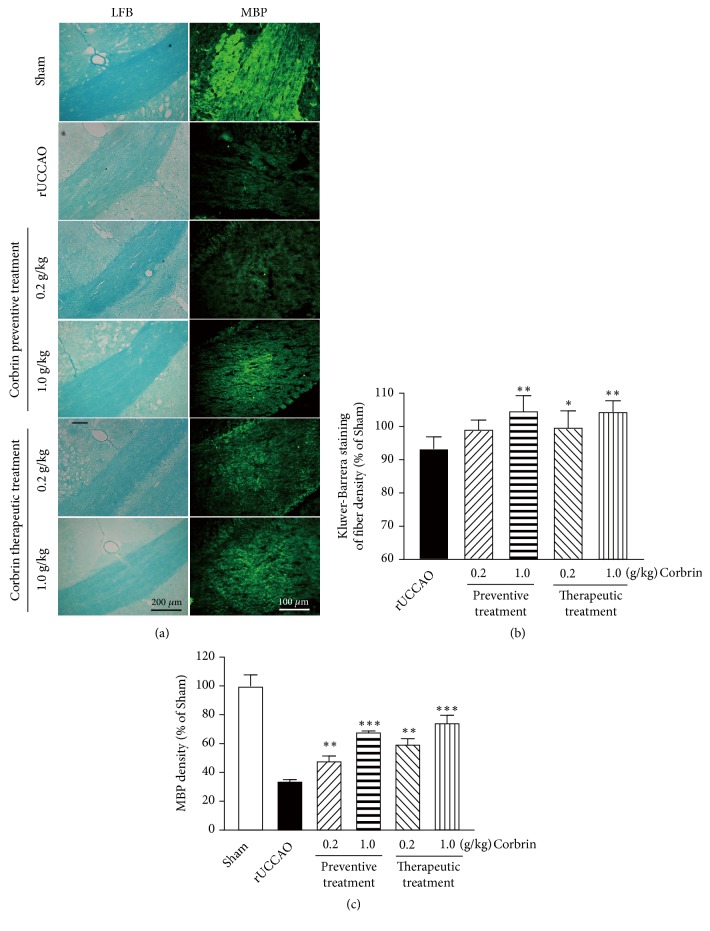
**The preventive and therapeutic effects of Cs-C-Q80 on fiber density and MBP expression in corpus callosum after rUCCAO injury.** (a) The Kluver-Barrera staining (left panel) and MBP immunostaining (right panel) were performed in indicated group. The representative images were shown. Semiquantification analysis of (b) fiber density and (c) MBP expression of indicated group were shown. Values are represented as mean ± SEM. *∗P*<0.05, *∗∗P*<0.01, and *∗∗∗P*<0.001* versus* the rUCCAO group; n=10.

Ten animals in each group were used for the luxol fast blue stain to discern white matter lesion in the corpus callosum. In the rUCCAO-operated mice, the fiber density in the corpus callosum irreversibly declined, which is a key pathological feature in vascular dementia. Both Cs-C-Q80 prophylaxis and treatment could obviously suppress white matter rarefaction in a dose-dependent way (Figures 4(a)-4(b)) as shown by the luxol fast blue stain. The expression of myelin basic protein (MBP) in the corpus callosum after rUCCAO sharply weakened to 37% of that in Sham group on Day 33. Prophylaxis with Cs-C-Q80 effaced the damage of rUCCAO on MBP expression to certain extent, while the therapeutic effect of Cs-C-Q80 showed slightly more extensive, but not statistic significant effects compared with the prophylactic treatment groups (Figure 4(c)). Treatment of Cs-C-Q80 alone for 28 days had no impact on the expression of MBP (data not shown).

### 3.4. Cs-C-Q80 Administration Alleviated rUCCAO-Induced Astrocyte Activation

**Figure 5:**
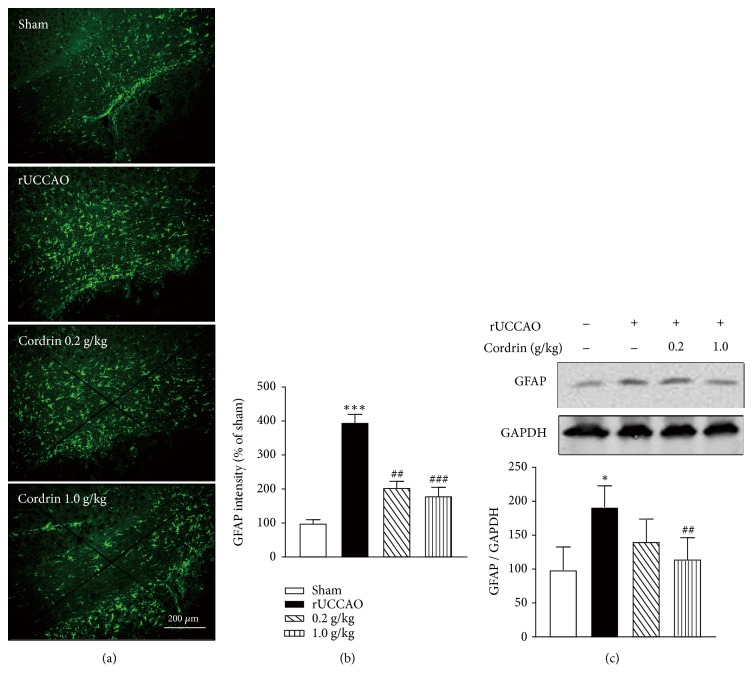
**The therapeutic effects of Cs-C-Q80 on astrocyte activation after rUCCAO injury.** Mice were subjected to rUCCAO for 28 days and during this period the indicated dosage of Cordrin was administrated. (a) The immunostaining of GFAP was shown and (b) the semiquantification analysis was presented. (c) The GFAP in corpus callosum was determined by Western blot and the bands intensity of GFAP normalized by GAPDH was shown in the columns. Values are represented as mean ± SEM. *∗P*<0.05* versus* the Sham group; ##*P*<0.01* versus* the rUCCAO group. For immunostaining, n=6; for Western blot, n=3.

The number of GFAP-positive astrocytes dramatically increased after rUCCAO modelling in the ipsilateral corpus callosum by immunohistochemical staining (Figures 5(a) and 5(b)). The expression of GFAP also upregulated 1.8-fold compared with the Sham (Figure 5(c)), suggesting the possibility of neuroinflammation. Cs-C-Q80 treatment by gavage for 28 days after rUCCAO operation dose-dependently inhibited the over activation of astrocytes by significantly decreasing either the number of astrocytes or the expression of GFAP in the corpus callosum.

### 3.5. Cs-C-Q80 Suppressed rUCCAO-Induced Increase in TNF-*α* and IL-1*β* Expression

It was reported that increasing production of proinflammatory cytokines including TNF-*α* and IL-1*β* was involved in the pathological process of ischemic injury, so the expression of TNF-*α* and IL-1*β* in the corpus callosum was examined by Western blot. Both the TNF-*α* and IL-1*β* level in the rUCCAO-operated mice elevated compared with the Sham (*P*<0.05). The 0.2 g/kg Cs-C-Q80 treatment only slightly downregulated the TNF-*α* and IL-1*β*, while 1.0 g/kg Cs-C-Q80 treatment significantly inhibited the TNF-*α* and IL-1*β* increasing (*P*<0.05), suggesting the involvement of the inflammation-resistant of Cs-C-Q80 treatment on vascular dementia.

## 4. Discussion

*Cordyceps sinensis* has been applied medicinally for over 2000 years in China with a variety of documented biological activities [5, 16–18]. However the beneficial effects of* Cordyceps sinensis* against brain disorders, in particular vascular dementia, have not been fully determined. In this study, we employed the mice model with chronic cerebral hypoperfusion induced by rUCCAO, which induced the impairment of white matter [2]. Our previous work identified that rUCCAO-treated mice showed significant damage of spatial working memory [19]. These features were in consistent with the clinical cognitive impairment in SIVD patients. Here we carried out the objects recognition test and Morris water maze test to examine the nonspatial working memory and spatial learning memory, respectively. The results showed that prophylaxis with either 0.2 g/kg or 1.0 g/kg Cs-C-Q80 one week before and 28 days after modelling attenuated rUCCAO-induced cognitive impairment (Figure 2). Since the imperceptibility of brain hypoperfusion and its consequent vascular dementia, it will be more valuable to evaluate the therapeutic effects of Cs-C-Q80 after the onset of ischemia. To this end, we set another group of experiment, in which Cs-C-Q80 was administrated for 28 days after rUCCAO. The results showed that postischemia treatment of Cs-C-Q80 rescued the rUCCAO-induced cognitive impairment, as revealed by the animal behavioral tests (Figure 3). The dosage of 1.0 g/kg Cs-C-Q80 daily significant prevented cognitive impairment in both administration manners, suggesting a promising dosage for clinical practice. According to the body surface area normalization [20], this dosage is approximately 4.8 g daily for an adult (60 kg of body weight), which is in the range of current prescriptions. Postsurgery administration of Cs-C-Q80 showed comparable efficacy in object recognition test and Morris water maze test with its pretreatment administration. This result suggested that pretreatment of Cs-C-Q80 might be prescribed as a therapeutic agent against vascular dementia. In addition, prophylactic administration, in particular the 0.2 g/kg dosage of Cs-C-Q80, showed a larger extent of protection in rescuing the spatial memory impairment. It is plausible that Cs-C-Q80 pretreatment may evoke the expressions of endogenous protective proteins in ischemic brains [21]. Cs-C-Q80 is fermented powder of the* Cordyceps sinensis*; previous studies showed that the chemical components of Cs-C-Q80 are similar to that of natural* Cordyceps sinensis*,

White matter is the tissue through which messages pass between different brain regions, as well as with peripheral nervous system like optic nerve. The white matter injury arises from stenosis and hypoperfusion involving multiple small arterioles. Pathological features of white matter lesions include diffuse myelin pallor, astrogliosis, rarefaction of the neuropil, widening of perivascular spaces, and loss of myelin and axons without definite necrosis [1]. In particular, oligodendrocytes and oligodendrocyte progenitor cells loss are typical features of white matter injury [4]. In addition, the white matter lesions could be taken as a predictor of cognitive decline and dementia [22, 23]. The white matter lesion by rUCCAO was reflected by KB staining. We found that either prophylactic or therapeutic application with Cs-C-Q80 effectively protected against the rUCCAO-induced white matter rarefaction (Figures 4(a) and 4(b)). MBP is an important protein responsible for the myelination of the nervous system. The decrease in the level of MBP was considered as an index for myelin degeneration during white matter lesion [24, 25]. We found Cs-C-Q80 significantly reversed the rUCCAO-induced MBP loss, indicating the promising role of Cs-C-Q80 in prophylaxis and treatment for white matter damage in SIVD (Figures 4(a) and 4(c)). Association between progression of white matter lesion and decline in cognitive performance has been established [26–29]. Our data suggested that Cs-C-Q80 may prevent cognitive impairment by alleviating the white matter injury. Some other disorders with the feature of axonal injury might also be attenuated with Cs-C-Q80 treatment, like spinal cord injury and glaucoma. Although Cs-C-Q80 showed a variety of potential medical applications, its underlying molecular targets have seldom been identified. Cordycepin, a component from* Cordyceps sinensis*, activated A3 adenosine receptor in bladder cancer cells [30] and inhibited EGFR in lung cancer cells [31]. Cordycepin also regulates several cell signaling pathways, including the PI3K/Akt, mTORC1, and AMPK [32, 33].* Cordyceps sinensis* extracts suppressed the secretion of inflammatory factors [34] and reduced oxidative stress [35]. Therefore, Cs-C-Q80 may confer its neuroprotection through a number of targets. Further studies are needed to elucidate the molecular targets of Cs-C-Q80 in ischemic brains. Cs-C-Q80 is fermented powder of the* Cordyceps sinensis. *Previous studies showed that the chemical components of Cs-C-Q80 are similar to that of natural* Cordyceps sinensis*, which contains amino acids, adenosine, and cordycepin [36]. It is currently not fully understood which components are bioactive and responsible for the benefits of Cs-C-Q80. It remains more unclear whether these components act alone or synergistically. These issues should be addressed in future studies.

**Figure 6:**
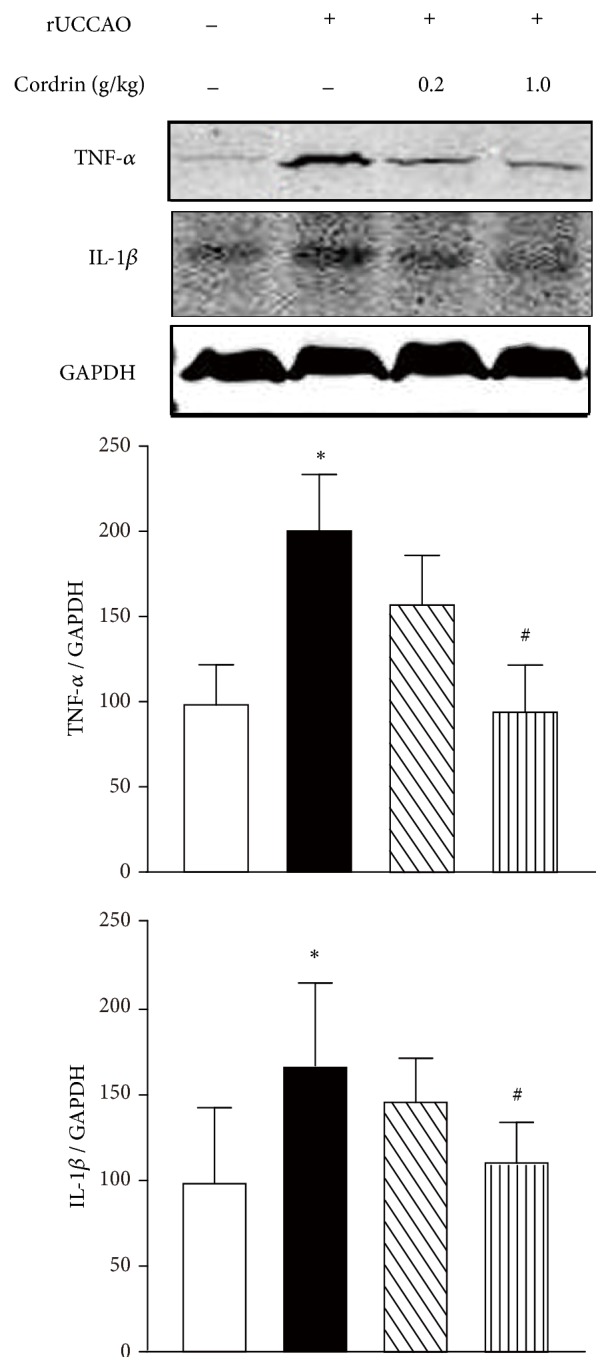
**The therapeutic effects of Cs-C-Q80 on TNF-** **α** **and IL-1** **β** **expression after rUCCAO injury.** The TNF-*α* and IL-1*β* expression were determined by Western blot and the lower columns represented semiquantification analysis of the bands intensity. Values are represented as mean ± SEM. *∗P*<0.05* versus* the Sham group; #*P*<0.05* versus* the rUCCAO group, n=3.

Chronic neuroinflammation in central nervous system is found to be involved in various neurodegenerative disorders, including vascular dementia [37, 38]. Neuroinflammation is detected in corpus callosum and results in white matter lesion and neuronal loss, leading to learning and memory impairment [2, 39, 40]. Among these, glia activation was one of the most important pathogenic mechanisms, which was observed in the white matter soon after the cerebral hypoperfusion in both human and experimental animals [41, 42]. Activated microglia and astrocytes are also considered to be the major sources of proinflammatory cytokines, including TNF-*α*, interleukin (IL)-1*β*, IL-6,* etc* [43]. Although the Cs-C-Q80 showed both the prophylactic and therapeutic effects against rUCCAO injury, given the imperceptibility of brain hypoperfusion, we focused on the anti-inflammatory effects of Cs-C-Q80 only by therapeutic administration. In Figure 5, either the number of astrocytes or the expression level of GFAP in the corpus callosum significantly boosted after rUCCAO, whereas taking Cs-C-Q80 for 28 days strongly repressed the activation of astrocytes in a dose-dependent manner. Furthermore, it was previously reported that the cerebrospinal fluid levels of TNF-*α* strikingly increased in vascular dementia and Alzheimer's disease patients [44]. Likewise, we found that the level of TNF-*α* also elevated after rUCCAO and can be effectively counteracted with Cs-C-Q80 treatment (Figure 6). In addition, IL-1*β* is highly relevant to hypoperfusion-induced dementia by impairing oligodendrocyte progenitor cells and thus lead to white matter injury [4]. We found that Cs-C-Q80 also downregulated the expression of rUCCAO-induced IL-1*β*. Similarly,* Cordyceps sinensis* was able to inhibit ischemia/reperfusion- (IR-) induced infiltration of polymorphonuclear cells and upregulation of the brain production level of C3 protein, IL-1*β*, NF-*κ*B, and other inflammatory mediators [8, 9]. Therefore, it was possible that Cs-C-Q80 defended the white matter lesions induced by rUCCAO via direct anti-inflammation.

## 5. Conclusions

In conclusion, our results demonstrated that both preventive and treatment administration with Cs-C-Q80 significantly improved the learning and memory deficit in the rUCCAO-induced SIVD mouse model. Cs-C-Q80 ameliorated the white matter lesions via direct or indirect anti-inflammatory actions. Therefore, Cs-C-Q80 could be a new promising therapeutic approach for preventing and treating vascular dementia.

## Data Availability

The data used to support the findings of this study are included within the article.
